# Arterial Tortuosity Syndrome in a Newborn: A Case Report With Literature Review

**DOI:** 10.7759/cureus.32899

**Published:** 2022-12-24

**Authors:** Sania Al-Blushi, Najwa Abdalkabeer A Bantan, Saad Al-Abdullatif, Mohiuddin M Taher

**Affiliations:** 1 Radiology, Maternity and Children’s Hospital, Dammam, SAU; 2 Radiology, Al Noor Specialist Hospital, Makkah, SAU; 3 Pediatrics, Maternity and Children’s Hospital, Dammam, SAU; 4 Medical Genetics, Umm Al-Qura University College of Medicine, Makkah, SAU; 5 Science and Technology Unit, Umm Al-Qura University, Makkah, SAU

**Keywords:** middle eastern countries, saudi arabia, tgfβ signaling pathway, glucose transporter 10, disorders of connective tissue, disorders of collagen and elastin, abnormalities of collagen and elastin, collagen maturation, slc2a10 gene, arterial tortuosity syndrome

## Abstract

Arterial tortuosity syndrome (ATS; OMIM #208050) is a sporadic, autosomal, recessively inherited genetic disorder. ATS primarily causes the tortuosity and elongation of large and medium-sized arteries; however, other skeletal manifestations include dysmorphic features, such as hyperextensible skin, hypermobile joints, and congenital contractures. The present article reports the case of a female neonate, who, at birth, exhibited abnormal facial features, hypermobility of joints, and abnormal physical appearance. The patient was diagnosed with ATS during the first week of life, based on computed tomographic scans. In addition, angiographic results demonstrated elongation and tortuosity of the aorta, which were further supported using the results of genetic analysis. Mutation analysis of the solute carrier family 2 member 10 (SLC2A10) genes (Entrez Gene: 81031) detected a homozygous pathogenic c.243C>G (p. Ser81Arg) variant (dbSNP: rs80358230) in this patient, which supports the clinical diagnosis of ATS. Following the initial diagnosis, further investigations into the family history were carried out, and the results demonstrated that the patient’s paternal grandmother and paternal aunt were also positive for ATS. The patient was subsequently referred to a tertiary care center for genetic counseling and further follow-up. Notably, carrier testing for at-risk relatives is recommended to identify family members that may be affected by this condition.

## Introduction

Arterial tortuosity syndrome (ATS) is a rare, autosomal, recessive connective tissue disorder. ATS was initially reported ~55 years ago in a 10-year-old female patient [1]. ATS is characterized by blood vessel abnormalities and is often characterized by twists and turns, known as tortuosity, of the arteries [2,3]. Numerous reports have demonstrated that both male and female patients are equally at risk of developing this syndrome. Beyens et al. have reported a male-to-female ratio of 27:22 in a retrospective characterization of 50 ATS patients’ symptoms [2]. The ATS typically appears during the early years of life, depending on complications that occur in infancy and the participation of several systems affected by this recessively inherited disorder [2,4]. From the analysis of a cohort of 50 new ATS patients and reviewing the 52 previously reported ATS patients’ data, the age at diagnosis for this disease was found to be 4.84 years (mean) and two years (median) [2]. However, adult cases have been diagnosed at 50 years and above [5-7], and prenatal/antenatal cases of ATS have also been reported [8].

Notably, tortuosity produces disruptions in the circulation of blood and augmented vessel wall shear stress, ultimately leading to atherosclerosis and the potential for a cerebrovascular stroke. Increased tortuosity results in chronic hypertension and the loss of stiffness of the vessel wall [2,3]. Moreover, ATS involves both large and medium-sized arteries. Patients with ATS exhibit an array of symptoms, or in very rare cases, they may appear without visible symptoms [2,3,5]. In the majority of patients with ATS, there is a high incidence of vascular issues, such as severe ventricular hypertrophy and valvular regurgitation, and in rare cases, atrial fibrillation has also been reported [6,9]. Vascular dissection, distortion, and stenoses of the pulmonary artery branches leading to systemic ventricular pressure, the presence of an aneurysm (abnormal bulging), and, in rare cases, aneurysmal dilation of the major intracranial arteries have also been reported [2,3,5]. Dysmorphic skeletal features, hyperextensible skin, hypermobile joints, and congenital contractures are distinctive manifestations of this disease [2].

The genetic etiology of ATS is associated with missense mutations in the solute carrier family 2 member 10 (SLC2A10) gene (locus 20q13.12; MIM 606145) [2,3]. This gene encodes a glucose transporter 10 (GLUT10) protein that is expressed in high amounts in the liver and pancreas. GLUT10 transports ascorbate, which plays a major role in connective tissue metabolism, acting as a cofactor for collagen and elastin biosynthesis reactions [10]. In ATS, the inactivation of GLUT10 due to mutations in SLC2A10 leads to defective synthesis of collagen and/or elastin [2,10]. Reduced hydroxylation of the prolyl and lysyl residues results in the weakening of large and medium-sized arteries, due to upregulation of the transforming growth factor-β (TGFβ) signaling pathway [2,11]. Investigations using large vessel biopsies demonstrated distorted and disintegrated elastic fibers in vascular tissue [3,12]. Symptoms of ATS are comparable to other connective tissue disorders, such as Loeys-Dietz syndrome (LDS), Marfan syndrome (MFS), Ehlers-Danlos syndrome (EDS), and autosomal recessive cutis laxa type 1 (ARCL1), and because of this finding, ATS sometimes can be misdiagnosed as other inherited disorders of the elastin network [9]. The identification of genetic defects in patients with connective tissue disorder is essential for a proper diagnosis, prognosis, and genetic counseling. Confirmation of "arterial tortuosity" by radiological investigations, and the identification of specific gene mutation responsible for this connective tissue disorder helps in the differential diagnosis of this syndrome. A rare case presented with ATS was previously diagnosed with EDS [7].

## Case presentation

The present article reports the case of a four-day-old female patient who was born full-term via an uncomplicated vaginal delivery, following premature rupture of membranes (PROM), to healthy consanguineous parents. The patient was born to a primigravida mother who exhibited thalassemia and gestational diabetes mellitus. Following PROM, the patient was admitted to the nursery for observation and laboratory work.

The patient was vitally and clinically stable, hypotonic, and hyper-mobile bilaterally in the upper limbs. The patient was not in respiratory distress and was not pale, jaundiced, or cyanosed. The patient exhibited pectus excavatum (sunken chest). Notably, the eyelids of the patient were weak, did not open as far as expected, and constantly covered part of the eyes (blepharophimosis) with a down-slanting palpebral fissure. The patient possessed a high-arched palate and deviated nose. The skin of the patient was hyperextensible (cutis laxa). A soft murmur was heard at the second intercostal space on the right side.

**Figure 1:**
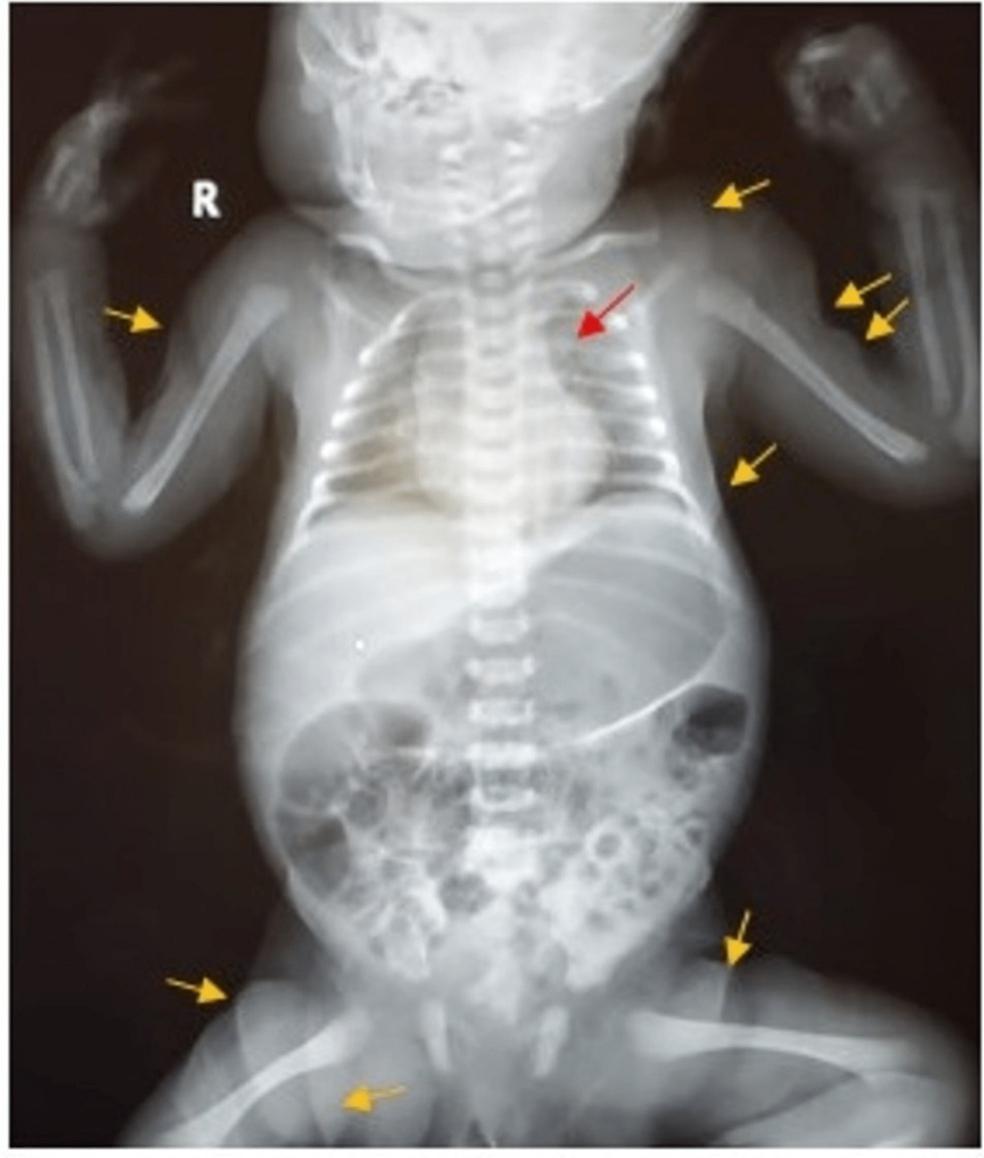
Frontal chest and abdomen X-ray of the four-day-old neonatal arterial tortuosity syndrome case showing down-slanted palpebral fissures and lax skin with bulging axillary skin folds (yellow arrows) and elongated tortuous aorta projected laterally (red arrow).

Following admission, basic laboratory tests were carried out, and complete blood counts (CBC) demonstrated a white blood cell count of 32 x 109/L (mainly neutrophils). A septic workup was carried out, and the patient was initially administered ampicillin and gentamicin. The results of the septic workup were negative, and antibiotics were subsequently stopped. Moreover, the results of the frontal chest radiograph in this four-day-old neonate demonstrated many down-slanted palpebral fissures and lax skin with bulging axillary skin folds due to skin hyperextensibility, and an elongated tortuous aorta projected laterally, which is not often visible in a young, healthy patient (Figure 1).

**Figure 2:**
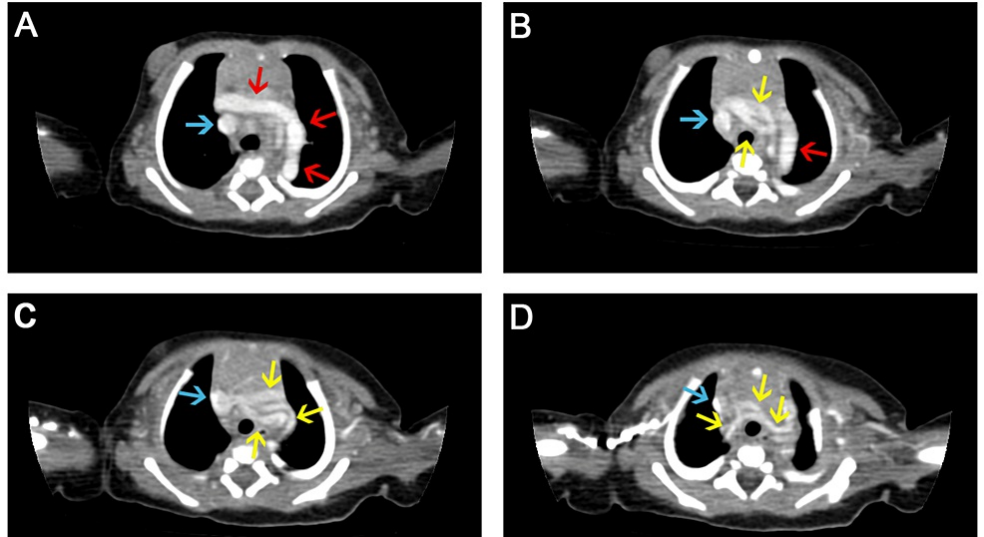
Multiaxial chest CT cuts (A-D) of the four-day-old neonatal arterial tortuosity syndrome patient showing elongation and tortuosity of the aortic arch (red arrows), proximal part of the great vessels (yellow arrows), and superior vena cava (blue arrows).

**Figure 3:**
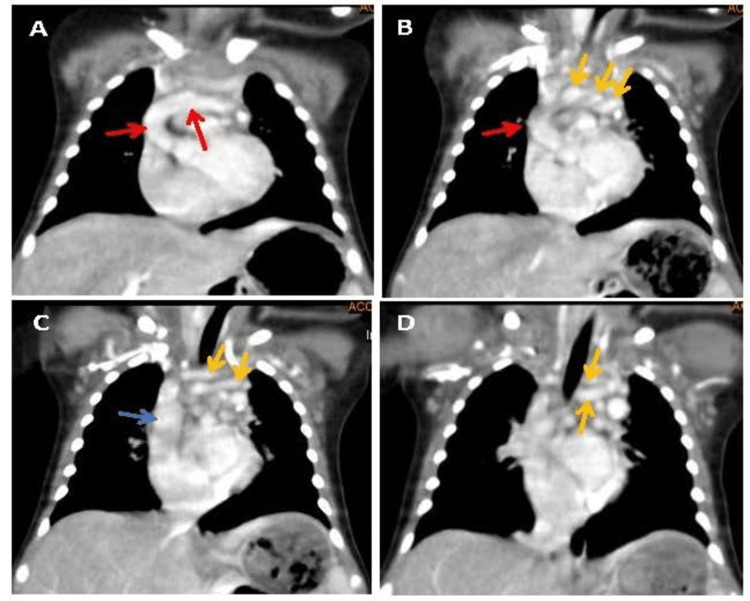
Maximal intensity projection (MIP) images (A-D) of the four-day-old neonatal arterial tortuosity syndrome case, in the coronal orientation of the chest, showing elongation and tortuosity of the aortic arch (red arrows), proximal part of the great vessels (yellow arrows), and superior vena cava (blue arrows).

Results of an echocardiogram demonstrated mild left ventricular hypertrophy, patent foramen ovale, an abnormal pulmonary artery course, patent ductus arteriosus and double aortic arch, healthy appearing cardiac chambers, no septal defect, no pulmonary artery drainage, no left or right ventricular outflow tract obstruction, and no pulmonary valve stenosis. Based on the results of the echocardiogram, a computed tomographic angiogram was requested. Results of the CT angiography demonstrated a left-sided aortic arch that appeared to be stretched, elongated, tortuous, and located laterally, indicative of aortic elongation (Figures 2, 3).

**Figure 4:**
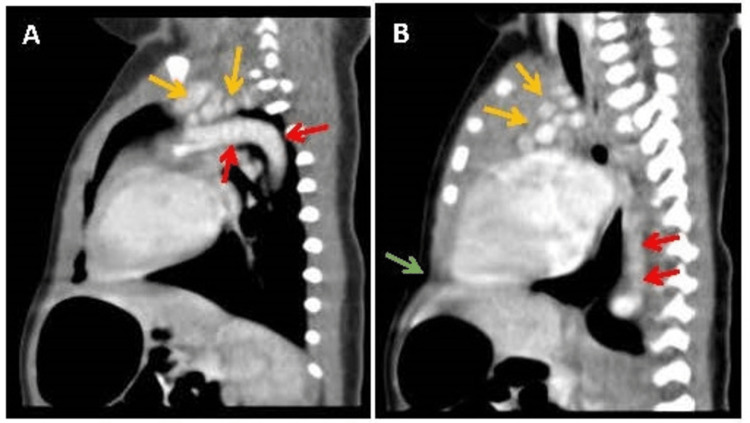
Maximal intensity projection (MIP) images (A and B) of the four-day-old neonatal arterial tortuosity syndrome case, in the sagittal orientation of the chest, showing elongation and tortuosity of the aortic arch (red arrows) and multiple rounded vascular structures in the superior mediastinum (yellow arrows), with pectus excavatum (green arrow).

In addition, tortuosity at the origins of the great vessel leads to a cluster of vessels in the superior mediastinum on a cross-section, indicative of a cluster of vessels (Figure 4). These results suggested that the patient was suffering from ATS. In addition, a depression below the sternum (pectus excavatum) was also noted on sagittal images (Figure 4B). Results of further imaging analyses, such as ultrasound examinations of the abdomen, head, and hip joints appeared healthy.

Sanger sequencing of all exons and intronic boundaries (+8 bp) of the SLC2A10 gene was performed in a commercial genetic laboratory at CGC Genetics, Porto, Portugal. Sequencing analysis revealed the presence of a pathogenic c.243C>G (p.Ser81Arg) homozygous variant in exon 2 of this gene. Notably, this result supported the clinical diagnosis of ATS. Following the initial diagnosis, further investigations into the family history were carried out. The parents of this case are first cousins, and the patient reported in this study is their first child. The father is slim built and tall and has dysmorphic facial features including a deviated septum, narrow maxilla, and narrow palpebral fissure, which are suggestive of an ATS carrier. The paternal grandmother and the paternal aunt also had such appearances; however, they do not have severe cardiovascular complications. It is reported that heterozygous carriers did not show severe vascular anomalies [3]. Following admission, the patient was fed orally and remained in stable condition until she was discharged seven days after birth. Following discharge, patient follow-up included appointments in the metabolic outpatient department (OPD) and cardiology OPD, and the results of these demonstrated no active issues. The patient was subsequently referred to a tertiary care center for genetic counseling and further follow-up.

## Discussion

The main characteristics of ATS include extension and tortuosity of the aorta and mid-sized arteries, and focal stenosis of a part of the pulmonary arteries and/or aorta [11]. These characteristics are coupled with features similar to a comprehensive connective tissue disease, including soft or doughy joint hypermobility and hyperextensible skin; diaphragmatic hernias and inguinal hernias [2,3,13,14], Morgagni hernias, and umbilical hernias have been reported in patients with connective tissue defects, including ATS [9,15]. Skeletal features consist of arachnodactyly, scoliosis, pectus excavatum (sunken chest) or pectus carinatum (protruding chest), knee/elbow contractures, and a finger or fingers that are fixed in a bent position at the inner joint, which cannot completely flatten (camptodactyly). Notably, severe complications in the cardiovascular system or ventricular hypertrophy resulting in global heart failure and ischemic events leading to organ infarction are the major cause of morbidity and mortality [2,11]. A mortality rate of 40% was reported in patients under five years [9]. There is an increased risk for respiratory insufficiency, ventricular hypertrophy resulting in global heart failure, progressive myocardiopathy, aneurysm development, and dissection, which can involve the aortic root and potentially entire arterial tree [2,9,11,16]. Moreover, patients with ATS are susceptible to vascular ischemia involving cerebrovascular circulation, which may cause a non-hemorrhagic stroke and infarctions in the abdominal arteries at any age. Takahashi et al. have reported that ATS is associated with early-onset pulmonary emphysema, indicating that some patients with ATS may also require close attention for chronic obstructive pulmonary disease [15].

Initially, ATS cases were mainly reported in Europe and Middle Eastern countries, and only sporadic reports were available from other countries. The reason could be this disease is underreported; however, recently, few reports have been published from India, South Korea, China, and Japan [8,15,17]. A single case of ATS presenting with epilepsy was also reported, indicating that some patients presenting with epilepsy might need serious cerebral vascular evaluation [17]. A case with severe tortuosity of cerebral arteries causing migraine headaches has also been reported, suggesting that further attention is needed to investigate the intracranial blood flow changes in ATS cases [16]. In another rare case, Naunheim et al. have observed persistent pulmonary hypertension and an association of venous tortuosity with ATS [5].

A total of 114 variants have been reported in the "Global Variome shared LOVD" (databases.lovd.nl/shared/genes/SLC2A10). There are 16 pathogenic variants causing ATS reported in the ClinVar database. The SLC2A10 gene contains five exons, and a majority of mutations are found in exon 2 and exon 3, and to a lesser extent few mutations are reported in exon 4, but so far, no mutations have been reported in exon 1 and exon 5. Nearly all reported missense variants were in the transmembrane or endofacial domains of the GLUT10 protein. It has been shown that all parents of affected individuals were heterozygous for SLC2A10 mutations [10,13], and heterozygous carriers did not show any vascular anomalies [3]. All patients diagnosed with ATS harbored homozygous or compound heterozygous mutations. The presence of homozygous and loss of function mutations in the SLC2A10 gene is responsible for ATS [10].

We have shown the spectrum of mutations found in the SLC2A10 gene in patients diagnosed with ATS from various published reports from different countries in Table 1. There are 24 missense, 10 nonsense (causing termination codon), 15 compound heterozygous variants, two variants with gross deletion of the full coding sequence, one variant with more than 40 amino acid deletion, 13 variants with single nucleotide deletion, two variants with small deletions of four bases, and one variant with two nucleotide deletions have been identified (Table 1). All deletion variants caused frameshift and termination codon. Also, one intronic splice variant causing exon skipping, and two intronic splice variants have been reported (Table 1). These variants were found in families from the Netherlands, Belgium, United Kingdom, France, Poland, USA, Turkey, Sudan, Spain, the Republic of Macedonian, Qatar, Saudi Arabia, Morocco, and Italy. From the Asian region, only one variant each from Japan, India, and China has been reported (Table 1). Six variants that are reported in the ClinVar database were not reported in published literature (Table 1).

**Table 1:** Previously published SLC2A10 mutations reported in arterial tortuosity syndrome patients. * Compound heterozygous mutation; # submission accession number. SLC2A10: solute carrier family 2 member 10; NA: not available.

Genotype	cDNA changed	Amino acid changed	Exon	Mutation type/functional consequence	dbSNP-ID	References
Heterozygous	c.4+3A>T*	N/A	Intron 1	Splice mutation	NA	[2]
	c.394C>T*	p. (Arg132TrpX)	2	Nonsense	rs121908173	
Homozygous	c.899T>G	p. (Leu300Trp)	2	Missense	rs771702107	[2]
Heterozygous	c.800delC*	p. (Ser268GlnfsX12)	2	Frameshift deletion-terminating	NA	[2]
	c.394C>T*	p. (Arg132TrpX)	2	Nonsense	rs121908173	
Homozygous	c.674G>A	p. (Arg225His)	2	Missense	rs34295241	[2]
Heterozygous	c.4+5G>A*	N/A	Intron 1	Splice mutation	NA	[2]
	c.1465G>C*	p. (Gly489ArgX)	4	Nonsense	rs201268555	
Homozygous	c.709G>A	p. (Gly237Arg)	2	Missense	rs1463765996	[2]
Homozygous	c.484delT	p. (Trp162GlyfsX83)	2	Frameshift deletion-terminating	NA	[2]
Heterozygous	c.314G>A*	p. (Arg105His)	2	Missense	rs753280877	
	c.727C>A*	p. (Gln243Lys)	2	Missense	rs1434246419	[2]
Homozygous	c.395G>A	p. (Arg132Gln)	2	Missense	rs376346077	[2]
Homozygous	c.68G>A	p. (Gly23Asp)	2	Missense	rs1979801533	[2]
Homozygous	c. 1333 del G	p. (Gly445GlufsX40)	3	Frameshift deletion-terminating	rs587776600	[6]
Homozygous	c.243C>G	p. (Ser81Arg)	2	Missense	rs80358230	[10,18]
Homozygous	c.318delT	p. (Ala106AlafsX138)	2	Frameshift deletion-terminating	NA	[19]
Heterozygous	c.1309G > A*	p. (Glu437Lys)	3	Missense	rs763220502	[20]
	c.1330C > T*	p. (Arg444X)	3	Nonsense	rs370547023	[20]
Homozygous	c.1309G>A	p. (Glu437Lys)	3	Missense	rs763220502	[11]
Heterozygous	c.1309G>A*	p. (Glu437Lys)	3	Missense	rs763220502	[3]
	c.1411+480_c.1547+299del*	p. (Gly471-Arg515delXfs)	4	Frameshift deletion-terminating	NA	
Heterozygous	c.1334delG*	p. (Gly445GlufsX40)	3	Frameshift deletion-terminating	rs587776600	[3]
	c.1411+480_c.1547+299del*	p. (Gly471-Arg515delXfs)	4	Frameshift deletion-terminating	NA	
Heterozygous	c.1309G>A*	p. (Glu437Lys)	3	Missense	rs763220502	[3]
	c.730_733delCTAA*	p. (Leu244GlnfsX35)	2	Frameshift deletion-terminating	rs864309481	
Homozygous	c.1411+1G>A	Intronic	Intron 2	Splice mutation, Exon 3 skipping	rs864309479	[7]
Homozygous	c.254T>C	p. (Leu85Pro)	2	Missense	rs754120063	[11,16]
Homozygous	c.510G>A	p. (Trp170X)	2	Nonsense	rs80358229	[10,14]
Heterozygous	c.417T > A*	p. (Tyr139X)	2	Nonsense	rs572620317	[15]
	c.692G > A*	p. (Arg231Gln)	2	Missense	rs771028960	
Homozygous	c.961delG	p. (Val321CysfsX71)	2	Frameshift deletion-terminating	rs587776599	[10]
Heterozygous	c.692G>A*	p. (Arg231Gln)	2	Missense	rs771028960	[3]
	c.1334delG*	p. (Gly445GlufsX40)	3	Frameshift deletion-terminating	rs587776600	
Homozygous	c.1334G>A	p. (Gly445GlufsX40)	3	Missense	rs753723351	[3]
Homozygous	c.1334delG	p. (Gly445GlufsX40)	3	Frameshift deletion-terminating	rs587776600	[4]
Homozygous	c.1334delG	p. (Gly445GlufsX40)	3	Frameshift deletion-terminating	rs587776600	[10]
Heterozygous	c.394C>T*	p. (Arg132Trp)	2	Missense	rs121908173	[3]
	c.1334delG*	p. (Gly445GlufsX40)	3	Frameshift deletion-terminating	rs587776600	
Heterozygous	c.1334delG*	p. (Gly445GlufsX40)	3	Frameshift deletion-terminating	rs587776600	[3]
	c.1276G>T*	p. (Gly426Trp)	2	Missense	rs121908172	
Homozygous	c.313C>T	p. (Arg105Cys)	2	Missense	rs767864243	[13]
Homozygous	c.685C>T	p. (Arg229X)	2	Nonsense	rs756457861	[3,16]
Heterozygous	c.1334delG*	p. (Gly445GlufsX40)	3	Frameshift deletion-terminating	rs587776600	[4]
	c.685C>T*	p. (Arg229X)	2	Nonsense	rs756457861	
Homozygous	c.737G>A	p. (Gly246Glu)	2	Missense	rs564317065	[3]
Heterozygous	c.1057_1058delCT*	p. (Lys353ThrfsX8)	2	Frameshift deletion-terminating	NA	[8]
	c.912 T > G*	p. (Cys304Trp)	2	Missense	NA	
Heterozygous	c.394C>T*	p. (Arg132Trp)	2	Missense	rs121908173	[3]
	c.1276G>T*	p. (Gly426Trp)	2	Missense	rs121908172	
Homozygous	c.425G>T	p. (Gly142Val)	2	Missense	rs864309480	[3]
NA	c.483delT	p. (Trp162GlyfsX83)	2	Frameshift deletion-terminating	SCV002158946^#^	Invitae submitter/ClinVar database
NA	c.485G>A	p. (Trp162X)	2	Nonsense	SCV002020699^#^	Perkin Elmer Genomics/ ClinVar database
NA	c.473_476delCTGG	p. (Ala158ValfsX86)]	2	Frameshift deletion-terminating	SCV002234760^#^	Invitae submitter/ClinVar database
NA	c.1424T>A	p. (Leu475X)	3	Nonsense	SCV002171737^#^	Invitae submitter/ClinVar database
NA	g. (? _46709717) _ (46733854_?) del	NA	NA	A gross deletion of the genomic region encompassing the full coding sequence	SCV000836931^#^	Invitae submitter/ClinVar database
NA	g. (? _46709727) _ (46733844_?) del	NA	NA	A gross deletion of the genomic region encompassing the full coding sequence	SCV001197081^#^	Invitae submitter/ClinVar database

The identification of the exact genetic defects related to the respective connective tissue disorder utilizing molecular pathology and genomic techniques has become routine for a proper diagnosis. Connective tissue diseases such as MFS, LDS, and ATS frequently exhibit the same clinical features, and differential diagnosis is often difficult without genetic testing. Genetic mutations and proteins involved in different inheritable connective tissue disorders are summarized in Table 2. Cardiovascular abnormalities are more common, and a high prevalence of arterial tortuosity was reported in LDS, MFS, and ATS patients. Depending on the different systems involved and their genetic manifestation, these disorders are further classified into major subgroups (Table 2). Only certain rare types of EDS include a predisposition for severe cardiovascular issues (such as vascular EDS). Beals syndrome has a specific heart defect known as mitral valve prolapse, and in some cases, mitral regurgitation was reported. In 46% of cases with 7q11.23 duplication, syndrome dilation of the ascending aorta was reported. Occipital horn syndrome (OHS), also known as X-linked cutis laxa (CL) or EDS type IX, which is a rare connective tissue disorder, arterial aneurysm, and cerebrovascular tortuosity along with bruisable skin, hernias, hyperextensible joints, and varicosities accompanied by mild neurologic impairment were reported in this disorder. Also, in CL type IB, aneurysmal dilation, elongation, tortuosity, and narrowing of the aorta and pulmonary artery are known. Generalized arteriopathy (narrowing of arteries) and arterial stenoses make up the large majority of cardiovascular issues in patients with Williams syndrome (WS), and supravalvular aortic stenosis was found in 80% of cases of WS. In other connective tissue diseases such as Shprintzen-Goldberg syndrome, epidermolysis bullosa, and osteogenesis imperfecta, well-known cardiovascular abnormalities, including arterial tortuosity, tend to be less common and less severe (Table 2). More details regarding these connective tissue disorders can be found in the National Organization for Rare Disorders (NORD) database (https://rarediseases.org).

**Table 2:** Genetic mutations and proteins involved in different inheritable connective tissue disorders. (A) Occipital horn syndrome, also known as Ehlers-Danlos syndrome type IX and X-linked cutis laxa. (B) Most of the cases are not inherited but de novo. (C) Most of the cases are de novo, and 25% are inherited. (S) Four major types. (H) There are 13 types of EDS. MOI: mode of inheritance; AD: autosomal dominant; AR: autosomal recessive; MACS: macrocephaly, alopecia, cutis laxa, scoliosis syndrome; XLR: X-linked recessive.

Name of the disorder		Clinical characteristics	Mutated genes		Protein defects		MOI
Loeys-Dietz syndrome (LDS)		Symptoms appear early in childhood, thoracic aorta aneurysm, blood vessel tortuosity, hypertelorism, bifid (split), or broad uvula. Arachnodactyly, scoliosis, pectus excavatum or pectus carinatum, dural ectasia, loose joints, craniosynostosis, blue sclerae, exotropia, and cervical spine malformation or instability.	LDS type 1	TGFBR1	Defective collagen synthesis and maturation		AD
			LDS type 2	TGFBR2			
			LDS type 3	SMAD3			
			LDS type 4	TGFB2			
			LDS type 5	TGFB3/ SMAD2			
Marfan syndrome (MFS)		Cardiovascular, skeletal, and ocular systems, overgrowth of the long bones of the arms and legs, scoliosis, excavatum/carinatum, ectopia lentis, myopia, aneurysm, dissection of the aorta, mitral valve prolapse, and aortic and mitral regurgitation.	FBN1, TGFBR2, and TGFBR1		Defects in fibrillin-1		AD
Beals-Hecht syndrome (BHS)/congenital contractural arachnodactyly (CCA)		Fixed flexion (contracture) of certain joints, arachnodactyly, and/or abnormally shaped ears, kyphoscoliosis, talipes equinovarus or clubfoot, ulnar deviation of the fingers, and an abnormally short neck. In extreme cases, ectopia lentis. In some cases, affected individuals may have a mitral valve prolapse.	FBN2		Defects in fibrillin-2		AD
Shprintzen-Goldberg syndrome		Craniofacial, skeletal, and cardiovascular deformities. Craniosynostosis, regurgitation or prolapse of the valves, and aortic root enlargement and aneurysm. Cardiovascular abnormalities tend to be less common and less severe.	SKI gene		Defects in SKI protein		AD/de novo mutations
Familial thoracic aortic aneurysms and dissections type 2 (TAAD2)		Aortic abnormalities include dilation, aneurysm, tear, or dissection near the heart. Aortic aneurysms can present as pain in the neck, jaw, back, or chest. Increased risk of heart attack or stroke. Tall and thin body, painful swallowing, flat feet, curved spine, long arms, legs, and fingers, or sinking chest or scoliosis, skin discoloration, and stretch marks.	FBN1, COL3A1		Defects in fibrillin-1 and type III procollagen		AD
Ehlers-Danlos syndrome (EDS)^H^		Articular hypermobility, elastic skin, excessive fragility of the skin, and small blood vessels. Only certain rare types of EDS include a predisposition for severe cardiovascular issues (such as vascular EDS); other types predominantly alter the skin and joints.	Arthrochalasia	COL1A1/COL1A2	Type I collagen		AD
			Cardiac-valvular	COL1A1	Type I collagen		AD
			Vascular type	COL3A1	Type III collagen		AD
			Classical	COL5A1	Type V collagen		AD
			Classical	COL1A1/COL5A2	Type I collagen		AD
			Myopathic	COL12A1	Type XII collagen		AD/AR
			Periodontal	C1R or C1R	Complement (C1r and C1s) pathway activation and connective tissue alterations		AD
			Classical-like	TNXB	Tenascin XB		AR
			Kyphoscoliotic	FKBP14/PLOD1	Lysyl hydroxylase 1 FKBP22		AR
Cutis laxa (CL)		Loose skin (lax), inelastic, abnormal skin may give a prematurely-aged appearance. Unlike similar skin disorders, easy bruising and scarring are generally not associated with cutis laxa. The joints are often abnormally loose (hypermobility) because of lax ligaments and tendons.	Type 3	ALDH18A1	Abnormal collagen and elastin synthesis		AR
			Type 2A	ATP6V0A2	Decreased secretion of elastin		AR
			OHS (A)	ATP7A	Abnormal collagen cross-linking		XLR
			Type I	ELN	Formation of amorphic clumps of elastic fibers		AD
			Type IB/EFEMP2-related	FBLN4	Abnormal elastin assembly		AR
			Type 2/type 1A	FBLN5	Abnormal elastic fibers		AD/AR
			Type IC	LTBP4	Abnormal elastic fiber assembly		AR
			Type 2B	PYCR1	Abnormal collagen and elastin synthesis		AR
			MACS syndrome	RIN2	Defective elastic tissue		AR
Osteogenesis imperfecta (OI)^(S)^		Bones are likely to break from mild to moderate trauma, loose joints and muscle weakness, fractures, hearing loss, and dentinogenesis imperfecta.	COL1A1 or COL1A2		Type 1 collagen		AD/AR
Epidermolysis bullosa (EB)		Blistering skin and mucosa in response to little or no apparent trauma. Rarely cardiomyopathy, genitourinary complications, and gastrointestinal issues. Nails may be thickened, arcuate, or herpetiform, blistering, and crusting on an inflammatory base. Tense blisters and healing erosions affect sites of friction on the feet. Plantar keratoderma and mottled hypo- and hyperpigmentation on the lower abdomen.	Dystrophic	Collagen VIIA1	Abnormality in collagen VII		AD/AR
			Simplex	KRT5 or KRT14	Keratin 5 or 14		AD/AR/de novo mutations
			Junctional	LAMA3, LAMB3, LAMC2, COL17A1, ITGA6, ITGB4, ITGA3	Laminin 332, type XVII collagen, integrin α6β4, integrin α3 subunit		AR
Arterial tortuosity syndrome (ATS)		Weak, long, tortious arteries are prone to aneurysms and tearing; blockages can lead to respiratory problems, heart attacks, strokes, or heart failure. Scoliosis, sunken or protruding chest, slender fingers and toes, and loose and flexible joints. Stretchable, soft skin. Hernias, diverticula or pouches in the intestinal walls, and keratoconus.	SLC2A10		Abnormal collagen and elastin		AR
Supravalvular aortic stenosis (SVAS)/elastin arteriopathy syndrome	Blood vessels are often significantly narrowed, including the aorta, and pulmonary artery reconstruction, poor muscle tone and lax joints, and problems with teeth.		ELN		Abnormal elastin		AD
Williams syndrome (WS)/Williams-Beuren syndrome (WBS)	The aorta and the pulmonary arteries are narrowed and supravalvular aortic stenosis. Babies born with Williams syndrome tend to grow slowly. Many experience feeding problems. They may have extended colic (irritability and crying). Poor muscle tone and lax joints. Small and widely spaced teeth.		Deletion of approx. 1.5 Mb (20-25 genes) on chromosome 7; ex., ELN, GTF2I, GTF2IRD1, and LIMK1			Abnormalities in elastin	AD/de novo mutations (B)
7q11.23 duplications syndrome	An enlarged dilated aorta can lead to dissection. Patent ductus arteriosus and septal defects. Anxiety disorders in children, social phobias, and autosomal dominant disorders. Macrocephaly that is flattened in the back (brachycephaly). Developmental problems, especially with speech and motor skills for crawling and walking in babies. Weak muscle tone with abnormal movements on one side of the body.		7q11.23 duplications1.5-1.8 Mb; duplication of ELN, MDH2, YWHAG, HSPB1, ZP3, SRCRB4D, DTX2, POMZP3.BAZ1B, LIMK1, CYLN2, GTF2I, and GTF2IRD1			Increased elastin protein	AD/de novo mutations (C)

In the present case report, the diagnosis was further verified via the presence of comprehensive arterial tortuosity and a homozygous pathogenic variant in the SLC2A10 gene, determined by DNA sequencing [13]. This homozygous missense variant found in the present case was also previously reported in Middle Eastern families, including 14 patients from Qatar [10,18]. This variant of SLC2A10 NM_030777.4: c.243C>G (p. Ser81Arg) was present in the third transmembrane domain of the facilitative glucose transporter, GLUT10 protein [3,10]. Previous ATS cases were reported in Saudi Arabia, and a total of seven cases were reported by Faiyaz-Ul-Haque et al. in two Saudi Arabian families. These patients exhibited severe tortuosity, dilatation and stenosis of arteries, and pulmonary hypertension [13]. A total of 48 patients from Riyadh and Doha with SLC2A10 mutations were also diagnosed with ATS [12,18]. Histopathologic and ultrastructural investigations of skin and tissue biopsy specimens of ATS patients revealed large spaces in the collagen fibers, elastic fiber fragmentation, and increased collagen deposition in both skin and vascular biopsies [2,12]. This indicated disorganization of the connective tissue bundles and reductions in the amount and texture of elastin fibers in the skin [2,10]. In addition, irregular internal elastic lamina, absence of myofilaments, and disordered medial smooth muscle cells with vacuolated cytoplasm were reported in small arteries in the skin of patients with ATS [12,18]. Reduced levels of elastin and disorganization of collagen fibrils in the skin contribute to the lax skin phenotype in patients with ATS [19,20].

## Conclusions

The present case study reported a patient from the eastern part of Saudi Arabia with ATS diagnosed in her first week after birth. Our finding of this previously reported recurrent missense variant of the SLC2A10 gene in c.243C>G, in the present case, suggests that mutation profiling by genomic analysis in families may reveal the common origin and shared ancestry. More studies are needed to establish the founder mutation nature of this variant in a larger population, which may help in the genetic screening of ATS. Neonates and infants including their parents, presenting with features described in the present case study, including lax skin, elongated tortuous aorta, aortic arch, proximal part of the great vessels and superior vena cava, and pectus excavatum, should be screened for genetic mutations indicative of ATS.

## References

[1] Ertugrul A (1967). Diffuse tortuosity and lengthening of the arteries. Circulation.

[2] Beyens A, Albuisson J, Boel A (2018). Arterial tortuosity syndrome: 40 new families and literature review. Genet Med.

[3] Callewaert BL, Willaert A, Kerstjens-Frederikse WS (2008). Arterial tortuosity syndrome: clinical and molecular findings in 12 newly identified families. Hum Mutat.

[4] Palanca Arias D, Ayerza Casas A, Clavero Adell M (2022). Arterial tortuosity syndrome (variants in SLC2A10 gene) in two pediatric patients in the same city of Spain: a case report. Bull Natl Res Cent.

[5] Naunheim MR, Walcott BP, Nahed BV, MacRae CA, Levinson JR, Ogilvy CS (2011). Arterial tortuosity syndrome with multiple intracranial aneurysms: a case report. Arch Neurol.

[6] Rodríguez-Capitán J, Macías-Benítez M, Conejo-Muñoz L, Cordero-Aguilar A, López-Salguero R, Pérez-Villardón B (2020). Arterial tortuosity syndrome: a late and unexpected diagnosis and description of a novel likely pathogenic mutation. Rev Esp Cardiol (Engl Ed).

[7] Castori M, Ritelli M, Zoppi N (2012). Adult presentation of arterial tortuosity syndrome in a 51-year-old woman with a novel homozygous c.1411+1G>A mutation in the SLC2A10 gene. Am J Med Genet A.

[8] Liang M, Wen H, Li S (2021). Two fetuses in one family of arterial tortuosity syndrome: prenatal ultrasound diagnosis. BMC Pregnancy Childbirth.

[9] Wessels MW, Catsman-Berrevoets CE, Mancini GM (2004). Three new families with arterial tortuosity syndrome. Am J Med Genet A.

[10] Coucke PJ, Willaert A, Wessels MW (2006). Mutations in the facilitative glucose transporter GLUT10 alter angiogenesis and cause arterial tortuosity syndrome. Nat Genet.

[11] Ritelli M, Chiarelli N, Dordoni C (2014). Arterial tortuosity syndrome: homozygosity for two novel and one recurrent SLC2A10 missense mutations in three families with severe cardiopulmonary complications in infancy and a literature review. BMC Med Genet.

[12] Faiyaz-Ul-Haque M, Mubarak M, AbdulWahab A (2022). Ultrastructure abnormalities of collagen and elastin in Arab patients with arterial tortuosity syndrome. J Cutan Pathol.

[13] Faiyaz-Ul-Haque M, Zaidi SH, Al-Sanna N (2009). A novel missense and a recurrent mutation in SLC2A10 gene of patients affected with arterial tortuosity syndrome. Atherosclerosis.

[14] Moceri P, Albuisson J, Saint-Faust M (2013). Arterial tortuosity syndrome: early diagnosis and association with venous tortuosity. J Am Coll Cardiol.

[15] Takahashi Y, Fujii K, Yoshida A, Morisaki H, Kohno Y, Morisaki T (2013). Artery tortuosity syndrome exhibiting early-onset emphysema with novel compound heterozygous SLC2A10 mutations. Am J Med Genet A.

[16] Kocova M, Kacarska R, Kuzevska-Maneva K (2018). Clinical variability in two Macedonian families with arterial tortuosity syndrome. Balkan J Med Genet.

[17] Chen S, Hong Y, Sherchan P, Zhang JM (2014). Epilepsy as the first presentation of arterial tortuosity syndrome in a young girl: a case report. Turk Neurosurg.

[18] Faiyaz-Ul-Haque M, Zaidi SH, Wahab AA (2008). Identification of a p.Ser81Arg encoding mutation in SLC2A10 gene of arterial tortuosity syndrome patients from 10 Qatari families. Clin Genet.

[19] Karakurt C, Koçak G, Elkiran O, Coucke PJ, Van Maldergem L (2012). Arterial tortuosity syndrome: case report. Genet Couns.

[20] Drera B, Guala A, Zoppi N, Gardella R, Franceschini P, Barlati S, Colombi M (2007). Two novel SLC2A10/GLUT10 mutations in a patient with arterial tortuosity syndrome. Am J Med Genet A.

