# Correction: Arterial Tortuosity Syndrome in a Newborn: A Case Report With Literature Review

**DOI:** 10.7759/cureus.c86

**Published:** 2023-01-03

**Authors:** Sania Al-Blushi, Najwa Abdalkabeer A Bantan, Saad Al-Abdullatif, Mohiuddin M Taher

**Affiliations:** 1 Radiology, Maternity and Children’s Hospital, Dammam, SAU; 2 Radiology, Al Noor Specialist Hospital, Makkah, SAU; 3 Pediatrics, Maternity and Children’s Hospital, Dammam, SAU; 4 Medical Genetics, Umm Al-Qura University College of Medicine, Makkah, SAU; 5 Science and Technology Unit, Umm Al-Qura University, Makkah, SAU

This article has been corrected at the request of the authors to replace Figures 3 and 4 with versions containing more accurately placed arrows.

